# Development and external validation of a simple nomogram for predicting apnea in children hospitalized with bronchiolitis

**DOI:** 10.3389/fped.2022.922226

**Published:** 2022-10-19

**Authors:** Qiuyan Xu, Linlin Li, Li Shen, Xia Huang, Min Lu, Chunxia Hu

**Affiliations:** ^1^ Department of Pediatrics, Suzhou Science / Technology Town Hospital, Suzhou, China; ^2^ Department of Respiratory Medicine, Children's Hospital of Nanjing Medical University, Nanjing, China; ^3^ Department of Pharmacy, Suzhou Science / Technology Town Hospital, Suzhou, China; ^4^ Department of Respiratory Medicine, Children's Hospital of Wujiang District, Children's Hospital of Soochow University, Suzhou, China

**Keywords:** apnea, children, bronchiolitis, prediction, nomogram

## Abstract

**Background:**

Apnea is one of the most life-threatening complications of bronchiolitis in children. This study aimed to determine early predictors of apnea in children hospitalized with bronchiolitis and develop a simple nomogram to identify patients at risk of apnea.

**Methods:**

This retrospective, observational study included children hospitalized with bronchiolitis in two hospitals in China. Demographic and clinical characteristics, laboratory results, pathogens, and pulmonary iconography results were recorded. A training cohort of 759 patients (one hospital) was used to identify early predictors of apnea during hospitalization. The least absolute shrinkage and selection operator (LASSO) regression analysis method was used to optimize variable selection. The nomogram was developed visually based on the variables selected by multivariable logistic regression analysis. Discrimination (concordance index, C-index), calibration, and decision curve analysis (DCA) were used to assess the model performance and clinical effectiveness.

**Results:**

A total of 1,372 children hospitalized with bronchiolitis were retrospectively evaluated, 133 (9.69%) of whom had apnea. Apnea was observed in 80 of the 759 patients with bronchiolitis in the training cohort and 53 of the 613 patients in the external validation cohort. Underlying diseases, feeding difficulties, tachypnea, retractions and pulmonary atelectasis in the training cohort were independent risk factors for apnea and were assembled into the nomogram. The nomogram exhibited good discrimination with a C-index of 0.883 (95% CI: 0.839–0.927) and good calibration. The DCA showed that the nomogram was clinically useful in estimating the net benefit to patients.

**Conclusion:**

We developed a nomogram that is convenient to use and able to identify the individualized prediction of apnea risk in patients with bronchiolitis. These patients might benefit from early triage and more intensive monitoring.

## Introduction

Bronchiolitis is a common lower respiratory tract infectious disease in young children, and it is the leading cause of hospitalization in the first 12 months of life worldwide (1). Although most patients with mild symptoms require only home-based nursing or outpatient treatment, some patients experience more serious manifestations, such as cyanosis, tachypnea, dyspnea, apnea, or extreme hypoxia, and require hospitalization or even transfer to the pediatric intensive care unit (PICU) for further life support treatment. In developed countries, infants with bronchiolitis account for 18% of all pediatric hospitalized children (2, 3). In a serial, cross-sectional study, approximately 2%–6% of hospitalized children with bronchiolitis required admittance to a PICU, and 2%–3% required invasive mechanical ventilation (3).

Apnea is a life-threatening complication in children with bronchiolitis and the leading cause of pediatric treatment in the PICU (4, 5). Considering that apnea is a serious complication that could increase the risk of adverse clinical outcomes and mortality, some studies have suggested that the risk of apnea in some young infants with bronchiolitis is sufficient for hospitalization, especially in the PICU, where the patient can be more closely monitored (6–8). In recent retrospective studies, the incidence of apnea in children with bronchiolitis ranged from 1.2% to 28.8% (5, 7–10). Due to its high incidence and poor clinical prognosis, early assessment and timely integration of the risk factors for apnea in children with bronchiolitis and screening of high-risk patients are particularly important. Clinicians must develop targeted prevention and control strategies to improve the prognosis and reduce the risk of death for these patients. In particular, there are few studies on early predictors of apnea in patients with bronchiolitis. Therefore, we aimed to summarize the clinical features of apnea in patients with bronchiolitis and to develop and validate a simple nomogram for predicting children with bronchiolitis who are at a high risk for the development of apnea.

## Methods

### Study design and populations

**Figure 1:**
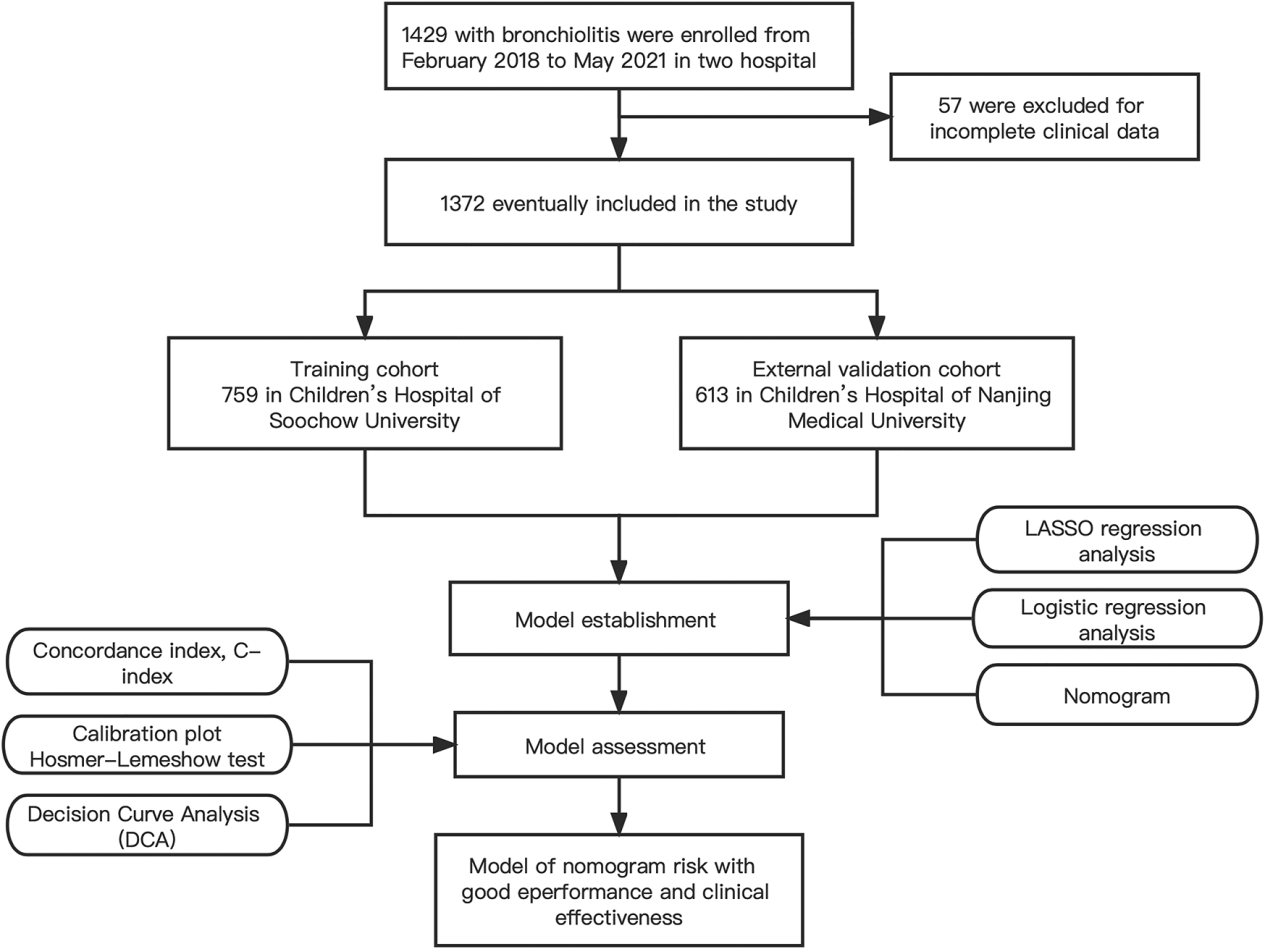
Flow diagram of study design.

This retrospective observational study was done in two hospitals designated for children hospitalized with bronchiolitis in the Chinese cities of Nanjing (Children's Hospital of Nanjing Medical University) and Suzhou (Children's Hospital of Soochow University) from February 2018 to May 2021. 31,549 patients with bronchiolitis visited the outpatient clinic or the emergency room, 1,429 of them were hospitalized for further treatment in two hospitals. A flow diagram of the study design is shown in Figure 1. This study was approved by the Ethics Committee of the Children's Hospital of Soochow University and Children's Hospital of Nanjing Medical University. Informed consent was obtained from the parents of all the children enrolled in this study.

Bronchiolitis is an acute lower respiratory tract infection characterized by rhinorrhea, cough, and diffuse wheezing and/or crackles (11, 12). Apnea is defined as the absence of airflow >10 s without (central apnea) or with the presence of persistent (obstructive apnea) or re-emerging (mixed apnea) respiratory efforts (13). We enrolled patients with the following inclusion criteria: patients ≤2 years of age who met the diagnostic criteria for bronchiolitis during hospitalization. Patients with incomplete clinical data or with evidence suggesting that wheezing was caused by tuberculosis and non-infectious factors, such as bronchial foreign bodies, were excluded.

The prediction model and nomogram development involved a cohort of patients from a single hospital (Children's Hospital of Soochow University). A second cohort from another hospital (Children's Hospital of Nanjing Medical University) was used for the external evaluation of the predictive performance of the model.

### Data collection and study outcomes

Data on the general features, personal history, laboratory results, clinical presentation, pathogens, and pulmonary iconography were recorded. General features included age, sex, and course of the disease before admission. Personal history, including preterm birth, bronchopulmonary dysplasia (BPD), congenital heart disease (CHD), apnea, severe malnutrition, and immunodeficiency was recorded as any underlying disease. Laboratory results included the white blood cell (WBC) count and C-reactive protein level. Clinical presentation included fever, cough, feeding difficulties, vomiting, oxygen, tachypnea, retractions, apnea, and crackles. The pathogens included *Mycoplasma pneumoniae*, bocavirus, respiratory syncytial virus, influenza viruses A and B, parainfluenza virus 1, 2 and 3, adenovirus, rhinovirus, and metapneumovirus. Pulmonary iconography revealed lung consolidation, pleural effusion, and pulmonary atelectasis. The primary outcome variable was apnea in children with bronchiolitis, who were divided into apnea and non-apnea groups according to whether apnea occurred during hospitalization.

### Statistical analysis

Descriptive continuous outcome variables are shown as medians with interquartile ranges (IQRs), and categorical variables are shown as whole numbers and proportions. Differences between continuous variables were assessed using the Mann–Whitney *U* test, and categorical variables were evaluated using the *χ*^2^ test or Fisher exact *χ*^2^ test. Training cohort data were used to develop the prediction model for the final logistic regression with apnea in hospitalized bronchiolitis patients as an outcome variable.

The least absolute shrinkage and selection operator (LASSO) method and cross-validation of the LASSO method, which are suitable for the regression of high-dimensional data (14, 15), were both used to select the optimal predictive features in risk factors from the hospitalized bronchiolitis patients. Features with nonzero coefficients in the LASSO regression model were selected (16) by using the “*glmnet*” package in R (http://www.r-project.org/). The features selected in the LASSO regression model were first analyzed using a univariate logistic model. Variables with a *p* value <0.05 were finally introduced into the multivariate logistic regression model. To provide clinicians with a quantitative tool to predict the individual probability of apnea, a nomogram based on the results of the multivariate logistic analysis was constructed using the “*rms*” package in R. The nomogram converted each regression coefficient in the multivariate logistic regression to a scale of 0 to 100 points in proportion. The effect of the variable with the highest *β* coefficient (absolute value) was specified as 100 points. The points across each variable were added to derive the total points and then converted to prediction probabilities.

Subsequently, we evaluated the predictive ability of nomogram discrimination and calibration. To evaluate model discrimination, we calculated the concordance index (C-index) using the “*Hmisc*” package. To analyze the consistency between the nomogram predictions and the actual observation results, calibration curves performed by using the “*rms*” package were created using 1,000 bootstrap resamples to decrease the overfitting bias. Using the “hoslem.test” function in the “*ResourceSelection*” package to perform the Hosmer–Lemeshow test to assess the calibration. To determine the net benefit of the model under the clinical threshold, decision curve analysis (DCA) was performed by using the “*rmda*” package.

Statistical analyses were conducted using the R software (version 4.1.1). Variables with a *p* value <0.05 were finally introduced into the multivariate logistic regression. In all analysis, a *p* value of less than 0.05 was considered to indicate statistical significance.

## Results

### Clinic characteristics

A total of 1,429 children hospitalized with bronchiolitis at two hospitals were enrolled in this study from February 2018 to May 2021. 57 of these patients were excluded because of incomplete clinical data. 1,372 patients were finally included in this study. The median age was 4.30 months (range 2.30–8.12), of which 90 (6.6%) had underlying diseases. The clinical features of patients with bronchiolitis mainly included fever (28.0%), cough (99.3%), apnea (9.7%), tachypnea(21.8%), retractions (10.3%) and feeding difficulties (18.5%). Furthermore, respiratory syncytial virus (RSV) (43.3%) appeared to be the dominant pathogen in patients, followed by rhinovirus (HRV) (25.8%), mycoplasma pneumoniae (MP) (15.8%).

In 133 of 1,372 (9.7%) patients with apnea, 75 (56.4%) were male and 58 (43.6%) were female, with a male to female ratio of 1.3 : 1. The ages of the patients ranged from 0.97 to 18 months. Among the 1,239 (90.31%) patients without apnea, 884 (71.3%) were male and 355 (28.7%) were female, with a male to female ratio of 2.5 : 1. The ages of the patients ranged from 1 to 24 months. The baseline characteristics of the study cohort are shown in Table 1. There were no significant differences between the training cohort and the external validation cohort in the distributions of demographic and disease characteristics.

**Table 1:** Demographic, clinical characteristics, and pathogens in patients with apnea and Non-apnea.

	Training cohort				External validation cohort				
	Overall (*n* = 759)	Apnea (*n* = 80)	Non-apnea (*n* = 679)	*p* *	Overall (*n* = 613)	Apnea (*n* = 53)	Non-apnea (*n* = 560)	*p* *	*p* **
**General features**									
Age (mouth) (IQR)	4.38 (2.33, 8.23)	3.50 (2.15, 7.11)	4.50 (2.43, 8.63)	0.007	4.00 (2.30, 7.83)	3.17 (1.87, 4.63)	4.34 (2.33, 8.00)	0.003	0.262
gender, male (%)	523 (69.4)	44 (55.0)	479 (71.1)	0.005	436 (71.1)	31 (58.5)	405 (72.3)	0.214	0.517
Course of disease before admission (day) (IQR)	6.00 (4.00,12.00)	4.00 (3.00, 7.25)	6.00 (4.00, 13.00)	<0.001	6.00 (4.00, 13.00)	4.00 (3.00, 8.00)	6.00 (4.1,14.00)	<0.001	0.609
**Personal history**									
Underlying diseases (%)	50 (6.6)	30 (37.5)	20 (2.9)	<0.001	40 (6.5)	22 (41.5)	18 (3.2)	<0.001	1
Preterm birth (%)	15 (2.0)	11 (13.8)	4 (0.6)		8 (1.3)	5 (9.4)	3 (0.5)		
BPD (%)	8 (1.1)	7 (8.8)	1 (0.1)		8 (1.3)	7 (13.2)	1 (0.2)		
CHD (%)	40 (5.3)	23 (28.8)	17 (2.5)		27 (4.4)	13 (24.5)	14 (2.5)		
Apnea history (%)	4 (0.5)	4 (5.0)	0 (0.0)		2 (0.3)	1 (1.9)	1 (0.2)		
Severe malnutrition (%)	3 (0.4)	2 (2.5)	1 (0.1)		1 (0.2)	1 (1.9)	0 (0.0)		
Immunodeficiency (%)	2 (0.3)	2 (2.5)	0 (0.0)		1 (0.2)	0 (0.0)	1 (0.2)		
**laboratory results**									
WBC count (×109/l) (IQR)	9.23 (6.92, 12.06)	8.81 (6.77, 12.45)	9.24 (6.98, 12.01)	0.721	9.32 (6.99, 12.33)	10.82 (7.38, 17.49)	9.26 (6.97, 12.13)	0.023	0.396
C-reactive protein (IQR)	0.83 (0.21, 3.06)	1.06 (0.09, 7.04)	0.78 (0.22, 2.95)	0.745	0.82 (0.23, 3.22)	1.12 (0.10, 7.00)	0.76 (0.23, 3.01)	0.392	0.800
**Clinic presentation**									
Fever (%)	203 (26.9)	17 (21.2)	186 (27.6)	0.282	180 (29.4)	42 (79.2)	138 (24.6)	<0.001	0.348
Cough (%)	751 (98.9)	79 (98.8)	672 (99.7)	0.212	611 (99.7)	53 (100.0)	558 (99.6)	1	0.626
Feeding difficulties (%)	134 (17.8)	40 (50.0)	94 (13.9)	<0.001	119 (19.4)	43 (81.1)	76 (13.6)	<0.001	0.48
Vomiting (%)	117 (15.4)	15 (18.8)	102 (15.0)	0.219	106 (17.3)	12 (22.6.)	94 (16.8)	0.281	0.349
Oxygen (%)	252 (33.4)	80 (100.0)	172 (25.5)	<0.001	188 (30.7)	52 (98.1)	136 (24.3)	<0.001	0.305
Tachypnea (%)	164 (21.8)	54 (67.5)	110 (16.3)	<0.001	134 (21.9)	40 (75.5)	94 (16.8)	<0.001	1
Retractions (%)	83 (11.0)	37 (46.2)	46 (6.8)	<0.001	58 (9.5)	24 (45.3)	34 (6.1)	<0.001	0.398
Crackles (%)	644 (85.4)	70 (87.5)	574 (85.2)	0.695	530 (86.5)	52 (98.1)	478 (85.4)	0.017	0.634
**Pathogens**									
MP (%)	120 (15.9)	8 (10.0)	112 (16.6)	0.171	96 (15.7)	8 (15.1)	88 (15.7)	1	0.957
BOKA (%)	89 (11.8)	5 (6.2)	84 (12.5)	0.148	–	–	–	–	–
RSV (%)	330 (43.8)	36 (45.0)	294 (43.6)	0.908	264 (43.1)	18 (34.0)	246 (43.9)	0.209	0.838
IVA (%)	2 (0.3)	0 (0.0)	2 (0.3)	1	2 (0.3)	0 (0.0)	2 (0.4)	1	1
IVB (%)	1 (0.1)	1 (1.2)	0 (0.0)	0.201	0 (0.0)	0 (0.0)	0 (0.0)	NA	1
PIV1 (%)	3 (0.4)	2 (0.3)	0 (0.0)	0.733	2 (0.3)	0 (0.0)	2 (0.4)	1	1
PIV3 (%)	57 (7.6)	5 (6.2)	52 (7.7)	0.806	54 (8.8)	6 (11.3)	48 (8.6)	0.673	0.458
ADV (%)	10 (1.3)	2 (2.5)	8 (1.2)	0.65	11 (1.8)	5 (9.4)	6 (1.1)	<0.001	0.632
HRV (%)	180 (23.9)	12 (15.0)	168 (24.9)	0.067	–	–	–	–	–
HMPV (%)	5 (0.7)	3 (3.8)	2 (0.3)	0.004	–	–	–	–	–
**pulmonary iconography**									
Lung consolidation (%)	43 (5.7)	13 (16.2)	30 (4.5)	<0.001	49 (8.0)	19 (35.8)	30 (5.4)	<0.001	0.116
Pleural effusion (%)	2 (0.3)	2 (2.5)	0 (0.0)	0.003	1 (0.2)	1 (1.9)	0 (0.0)	0.141	1
Pulmonary atelectasis (%)	11 (1.5)	7 (8.8)	4 (0.6)	<0.001	11 (1.8)	7 (13.2)	4 (0.7)	<0.001	0.784

Characteristics were summarized as median (IQR) or frequency (%). BPD, bronchopulmonary dysplasia; CHD, congenital heart disease; MP, mycoplasma pneumoniae; BOKA, bocavirus; RSV, respiratory syncytial virus; IVA, influenza virus A; IVB, influenza virus B; PIV1, parainfluenza virus 1; PIV3, parainfluenza virus 3; ADV, adenovirus; HRV, rhinovirus; HMPV, metapneumovirus.

* *p* value for difference between patients with Apnea and Non-apnea.

** *p* value for training cohort vs. validation cohort for overall characteristics.

In the training cohort, 759 patients were eligible for inclusion, including 80 with and 679 without apnea. Patients with apnea were younger than those without apnea. The course of the disease before admission was significantly shorter in patients with apnea than those without apnea. In terms of personal history, underlying diseases were more frequent in patients with apnea than in those without apnea. Patients with apnea had more severe clinical symptoms and were significantly more likely to have feeding difficulties (50%), tachypnea (67.5%), retractions (46.2%), lung consolidation (16.2%), pleural effusion (2.5%) and pulmonary atelectasis (8.8%) than those without apnea (13.9%, 16.3%, 6.8%, 4.5%, 0%, 0.6%, respectively, *p* < 0.05 for all comparisons). In terms of pathogens, apnea patients with HMPV were more than non-apnea patients (*p* < 0.05), the distribution of other pathogens between patients with and without apnea was no statistical significance, all these as shown in Table 1.

### Feature selection

**Figure 2:**
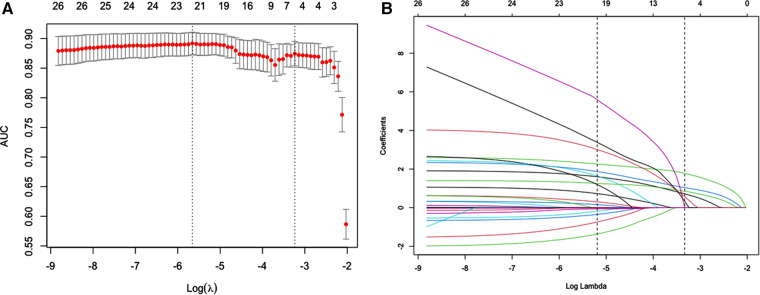
Possible risk factors for apnea using the LASSO binary logistic regression model and selection for using the least absolute shrinkage. (**A**) Optimal parameter (*λ*) selection in the LASSO model used 10-fold cross-validation *via* minimum criteria. The predicted AUC from the LASSO regression cross-validation procedure was plotted as a function of log (*λ*). The *x*-axis and *y*-axis represent the log (*λ*) and predicted AUC, respectively. The red dots represent the average predicted AUC for each model with a given *λ*. Vertical lines were drawn at the optimal values by using the minimum criteria and the 1 standard error (SE) of the minimum criteria (the 1-SE criteria). A *λ* value of 0.039 with log (*λ*) = −3.237 was chosen (1-SE criteria). (**B**) LASSO coefficient profiles of the 26 features. A coefficient profile plot was produced against the log (*λ*) sequence. Vertical line was drawn at the value selected using 10-fold cross-validation, where the 6 resulting features (1-SE criteria) with nonzero coefficients are indicated in the plot.

Of general features, personal history, clinic presentation, laboratory results, pathogens and pulmonary iconography characteristics, 26 features were reduced to 6 potential predictors on the basis of 759 patients in the training cohort (Figure 2) and were with nonzero coefficients in the LASSO logistic regression model. These 6 features including underlying diseases, feeding difficulties, tachypnea, retractions, pleural effusion and pulmonary atelectasis (Table 2).

**Table 2:** Factors associated with apnea in final multivariate regression model of training cohort.

Variable	Univariable models		Multivariate models	
	OR (95% CI)	*p*	OR (95% CI)	*p*
Underlying diseases	19.620 (10.486–37.525)	<0.001	14.086 (6.656–30.5635)	<0.001
Feeding difficulties	6.170 (3.781–10.091)	<0.001	4.590 (2.481–8.547)	<0.001
Tachypnea	10.649 (6.454–17.981)	<0.001	4.381 (2.295–8.426)	<0.001
Retractions	11.747 (6.910–20.068)	<0.001	4.575 (2.325–9.049)	<0.001
Pleural effusion	NA	0.979	–	–
Pulmonary atelectasis	12.83 (3.996–44.323)	<0.001	17.309 (3.482–82.878)	<0.001

### Logistic regression analysis and nomogram of apnea risk in patients with bronchiolitis

**Figure 3:**
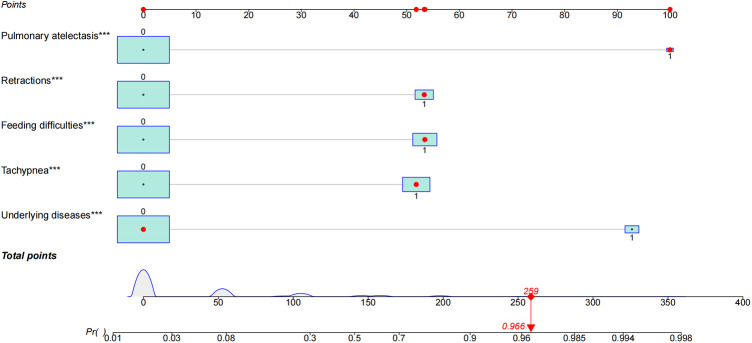
Characteristics in the nomogram to predict probability of apnea in patients with bronchiolitis. A nomogram for apnea in patients with bronchiolitis during hospitalization was developed and integrated with the predictors. Find the predictive points corresponding to each patient variable on the highest point scale and then add them to the total score points. The total points projecting to the bottom scale indicated the percentage of probability of apnea in hospitalized patients with bronchiolitis. The red dots in nomogram represent an example. The significance of the asterisks beside each variable represent importance of all the risk factors.

Pleural effusion was not identified as a significant predictors in the univariate logistic regression analysis. Other features including underlying diseases, feeding difficulties, tachypnea, retractions and pulmonary atelectasis were identified as significant predictors for apnea (Table 2). A multivariate logistic model containing the above five significant predictors together was established and presented as a nomogram (Figure 3). For example, using the nomogram, a patient with feeding difficulties, tachypnea, retractions and pulmonary atelectasis has an estimated probability of apnea of 96.6% (Figure 3).

### Nomogram model performance

**Figure 4:**
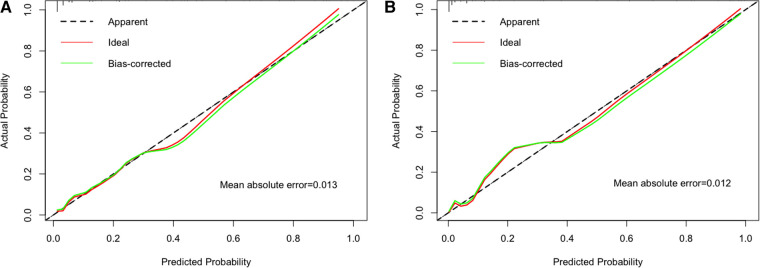
The calibration method with bootstrapping was used to illustrate the association between actual apnea and predicted apnea. Calibration plots showed the apparent (actual), bias-corrected (adjusted), and ideal (100% agreement) curves with bootstrapping samples. Bootstrapping involved 1,000 repetitions. Nomogram-predicted probability of apnea was plotted on the *x*-axis; the observed probability of apnea was plotted on the *y*-axis. The diagonal dotted line represents a perfect prediction by an ideal model. The solid line represents the performance of the nomogram, of which a closer fit to the diagonal dotted line represents a better prediction. (**A**) Training cohort; (**B**) external validation cohort.

The ability of final model was assessed to discriminate apnea in patients using C-index. The C-index of the nomogram for predicting apnea in the training cohort and external validation cohort were 0.883 (95% CI: 0.839–0.927) and 0.954 (95% CI: 0.924–0.984), which were significantly higher than the C-index obtained for each variable in the model (Supplementary Table S1). Good calibration was observed for the probability of apnea. The Hosmer–Lemeshow test confirmed that the prediction deviation was not significantly different between the risk prediction value of the nomogram and the actual observation value (*χ*^2 ^= 0.443, *p* = 0.999 in the training cohort; *χ*^2 ^= 5.522, *p* = 0.701 in the external validation cohort). In addition, the calibration curve of the nomogram for the prediction of apnea risk in patients with bronchiolitis demonstrated adequate agreement in the training and external validation cohorts (mean absolute error was 0.013 in training cohort and 0.012 in external validation cohort) (Figure 4), showing adequate agreement of the predictive nomogram with actual observations.

### Clinical usefulness of the nomogram

**Figure 5:**
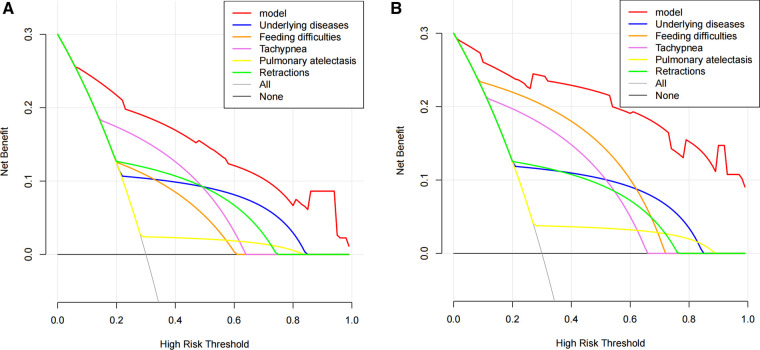
Decision curve analysis (DCA) of the apnea nomogram and single predictors. DCA was used to evaluate the clinical utility of the nomogram (17, 18). The y-axis indicates the net benefit. The red line represents the apnea nomogram. The grey line indicates the presumption that all patients had apnea. The black line indicates the presumption that no patients had apnea. The net benefit was estimated by subtracting the proportion of all patients with false positives from the true positive proportion, weighted by the relative harm of not obtaining treatment in comparison to the negative outcomes of an unnecessary treatment (14, 19). The decision curve indicated that if the threshold probability of a patient or doctor is 10% using the nomogram to predict the apnea in patients with bronchiolitis, it could add a greater advantage than the treat-all-patients scheme or the treat-none scheme. (**A**) Training cohort; (**B**) external validation cohort.

The benefit derived from applying the nomogram in clinical practice was assessed by the DCA (Figure 5). The DCA curve showed that threshold probabilities for the standardized net benefit associated with application of the nomogram in detecting apnea ranged from 0.08 to 1.0 in the training cohort and 0.02 to 1.0 in the external validation cohort. Within this range, the predictive performance of the nomogram was better than that of any single predictor, suggesting that the nomogram model has better clinical applicability.

## Discussion

In this study, we developed and verified a nomogram for the early prediction of apnea risk in patients with bronchiolitis. The nomogram, based on underlying diseases, feeding difficulties, tachypnea, retractions, and pulmonary atelectasis, had a discriminatory power (C-index) of 0.883 (95% CI: 0.839–0.927) in predicting the risk of apnea and had good clinical applicability. Apnea in children with bronchiolitis tends to have a poor prognosis and a high risk of death. Thus, a comprehensive assessment of the risk factors for apnea and early prediction of the risk of apnea in these patients have important clinical value by helping clinicians to identify high-risk patients, make targeted clinical decisions, and appropriately allocate critical care resources.

We found a 9.69% rate of apnea among hospitalized patients with bronchiolitis, our results were basically consistent with previous reports of range from 1.2% to 28.8% (5, 7, 9, 10). The observed difference in rate of apnea in different studies likely results from selection of different study populations. Clinical manifestations of bronchiolitis range from mild respiratory distress to incipient respiratory failure. Apnea is a potentially life-threatening complication of bronchiolitis. In younger children, especially infants younger than six weeks, apnea may be the only visible sign, sometimes in the absence of other clinical features of bronchiolitis (9, 20). Recently, several studies have reported predictors of apnea in patients with bronchiolitis (5, 7, 8). Ramos-Fernández et al. suggested that birth by cesarean section, fever, low weight, history of apnea, and severe bacterial infections were factors predictive of apnea in children with bronchiolitis (8). Willwerth et al. reported that children were considered to be at a high risk for apnea if they were at full-term birth but less than one month of age, had preterm birth (gestational age less than 37 weeks), were aged less than 48 weeks, or had a history of apnea (7). Different study designs and research subjects lead to differences in the research prediction results. In this study, the prediction of underlying diseases was consistent with the results of the above studies. If patients had underlying conditions, including preterm birth, BPD, CHD, a history of apnea, severe malnutrition, and immunodeficiency, the weight of the influence of a nomogram score of more than 90 on the risk of apnea could be increased, suggesting that they were especially important predictors of apnea.

Severe bronchiolitis in younger infants is more likely to present with retraction and feeding difficulties (21). Tachypnea, retraction, and low oxygen saturation were evaluated as severe bronchiolitis (22). In a prospective multicenter study of hospitalized children with bronchiolitis, apnea was not only associated with younger corrected age, lower birth weight, history of apnea, low or high respiratory rates, and low indoor air oxygen saturation was also risk factors for apnea (5). Similarly, the results of this study showed that patients with apnea had higher feeding difficulties, tachypnea, and retractions, which were predictors of apnea risk in patients with bronchiolitis. In our study, age was not a predictor for apnea in bronchiolitis, although bronchiolitis patients with apnea was significantly younger than that of the non-apnea group, which was different from the study Schroeder AR et al.. This result may be partly due to differences in the methods used to screen for risk factors in previous studies, and partly because we did not further subgroup analysis by age. RSV infection can cause severe bronchiolitis in younger children and is a common cause of hospitalization (23), which was confirmed in our study. A previous study reported that apnea was noted in approximately 9% of children hospitalized with RSV bronchiolitis and in 20% of those requiring intensive care support (24). Different from the admission criteria of our study, in the study of Wilson DF et al., the subjects were mainly infants ≤ 1 year-old hospitalized for bronchiolitis or RSV pneumonia, and 1/3 of them required further treatment in PICU, which could explain why the incidence of apnea in patients with bronchiolitis infected by RSV in this study(10.9%) was lower. In addition, we found that the prevalence of RSV infection between patients with and without apnea was not significantly different, and RSV infection was not a predictor of apnea, our results was consistent with the study of Schroeder et al. (5).

Previous studies have indicated the probability of pulmonary atelectasis in hospitalized patients with bronchiolitis was ranged from 14.3% to 42% (25–28), and about 50% of pulmonary atelectasis required further treatment in PICU (25, 26, 28). Although the occurrence of pulmonary atelectasis increased the risk of respiratory failure and mechanical ventilation, pulmonary atelectasis was not a risk factor for apnea in children with bronchiolitis (25). However, our study is different from previous studies. In this study, the incidence of pulmonary atelectasis in hospitalized children with bronchiolitis was significantly lower than that previous studies. Meanwhile, the probability of pulmonary atelectasis in patients with apnea was significantly higher than that of Non-apnea patients, and it was an independent risk factor for apnea. The main reasons for the differences in results may be due to the difference in the standards of hospitalization, the timing of chest imaging examinations, and race. These requires more multi-center, large-sample, prospective studies to further confirm.

Although most of the risk factors related to the occurrence of apnea in patients with bronchiolitis are consistent and similar to those reported in previous studies, this study is different from previous studies in the perspective of statistical research methods. Previous studies always used a single factor analysis to validate a multivariate analysis or performed stepwise regression analyses of the process to obtain the results. However, in this process, various confounding factors might be considered as variables along with the problem of multicollinearity. In this study, LASSO regression analysis was used to select the optimal predictive features, which provided a better solution to reduce multicollinearity and provided more accurate results, and then a traditional logistic regression analysis was also performed. A nomogram is a simple and intuitive expression of statistical model analysis results, which makes the prediction model more concise and effective in quantifying risks and has a higher application value. This advantage gives the nomogram more attention and application in medical research and clinical practice (7, 29, 30). At present, this study is the first to establish an early predictive nomogram model of apnea in patients with bronchiolitis. C-index, calibration and DCA curves were constructed for the training and validation sets to verify the accuracy, stability and clinical applicability of the model.

Our study has several potential limitations. First, our model was only developed for two hospitals in Jiangsu Province, with fewer samples and limited sample sources. Second, our study is a retrospective study, and the research conclusion still needs to be verified by further prospective multicenter large-sample cohort studies. Third, five variables were selected to predict the risk of apnea. They were derived from clinical data and easy to collect and analyze, which ensured the simplicity and practicability of the prediction model. However, innovative research indicators such as gestational age, feeding pattern, perinatal infection and birth weight for predicting the occurrence of apnea are lacking in this study. Therefore, we plan to conduct prospective cohort studies with more innovative indicators to improve the prediction performance of the apnea prediction model in the future.

In conclusion, this study presents a nomogram including underlying diseases, feeding difficulties, tachypnea, retractions and pulmonary atelectasis that is convenient to use and able to identify the individualized prediction of apnea risk in patients with bronchiolitis. These patients might benefit from early triage and more intensive monitoring to improve the prognosis.

## Data Availability

The raw data supporting the conclusions of this article will be made available by the authors, without undue reservation.
