# Supplementary material for: Development and external validation of a simple nomogram for predicting apnea in children hospitalized with bronchiolitis
Source: Front Pediatr. 2022 Oct 19;10:922226. doi: 10.3389/fped.2022.922226 (PMC9627176; doi:10.3389/fped.2022.922226)
Supplement: Supplementary file 1 [file DataSheet1.docx]

Supplement material

Method

Respiratory Pathogens
Direct immunofluorescence was used to detect respiratory syncytial virus (RSV); adenovirus (ADV); influenza virus A (IV A) and B (IV B); A D^3^ Ultra Respiratory Virus Screening and LD Kit (Diagnostic Hybrids, Athens, OH, USA) was used to identified parainfluenza virus 1, 2, 3 (PIV 1, 2, 3). Five or more inclusion bodies analyzed under fluorescence microscopy were defined as positive. Polymerase chain reaction (PCR) (nucleic acid amplification fluorescent reagent kit, Ann Gene Co., Guangdong, China) was used to detect Mycoplasma pneumoniae (MP), human metapneumovirus (HMPV), human bocavirus (HBoV), human rhinovirus (HRV) and according to the manufacturer’s instructions.

Supplement table 1. C-statistics for the nomogram and model variables in the training and external validation cohorts

|  | Training cohort | | | External validation cohort | | |
| --- | --- | --- | --- | --- | --- | --- |
|  | c-index | 95% CI | P | c-index | 95% CI | P |
| nomogram | 0.883 | 0.839–0.927 | - | 0.954 | 0.924–0.984 | - |
| Underlying diseases | 0.673 | 0.619-0.726 | ＜0.001 | 0.692 | 0.624-0.759 | ＜0.001 |
| Feeding difficulties | 0.680 | 0.624-0.737 | ＜0.001 | 0.838 | 0.783-0.893 | ＜0.001 |
| Tachypnea | 0.756 | 0.702-0.809 | ＜0.001 | 0.793 | 0.733-0.854 | ＜0.001 |
| Retractions | 0.697 | 0.641-0.753 | ＜0.001 | 0.696 | 0.628-0.764 | ＜0.001 |
| Pulmonary atelectasis | 0.540 | 0.509-0.571 | ＜0.001 | 0.563 | 0.516-0.609 | ＜0.001 |


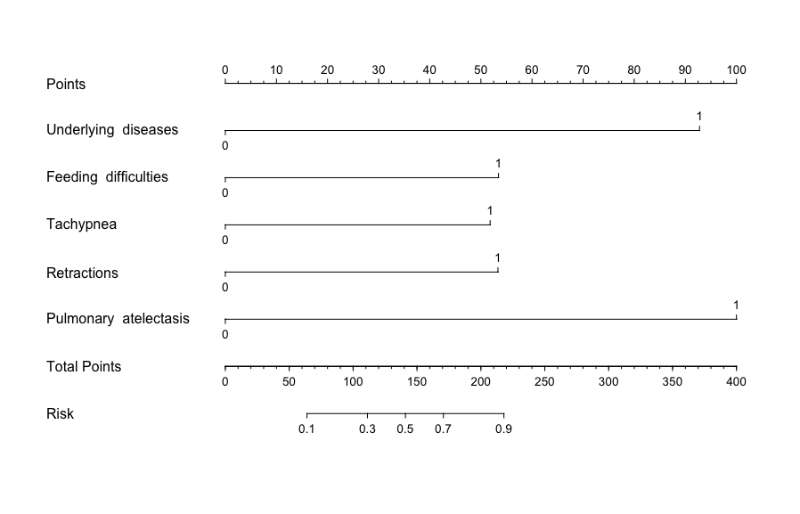

Figure E1. Characteristics in the nomogram to predict probability of apnea in patients with bronchiolitis

A B

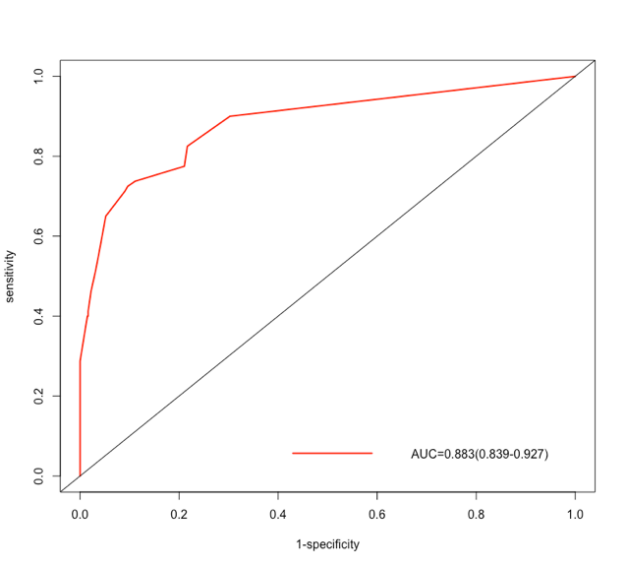

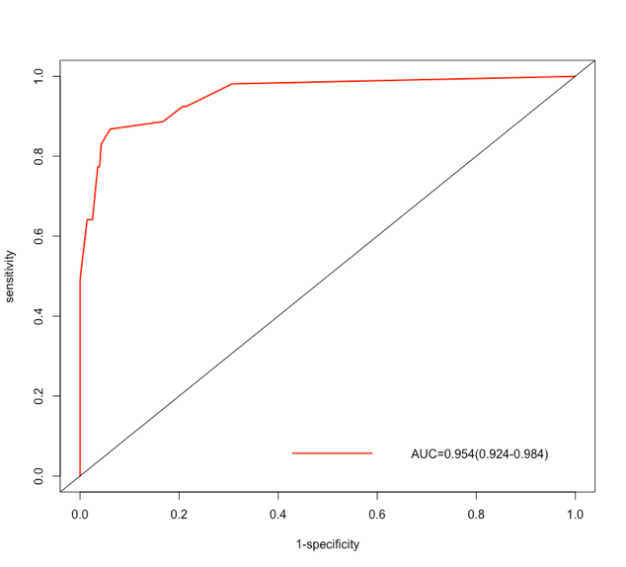


Figure E2. Receiver operating characteristic curve in the training cohort (A) and the in the validation cohort (B)
